# A green-synthesized phosphorescent carbon dot composite for multilevel anti-counterfeiting†

**DOI:** 10.1039/d1na00252j

**Published:** 2021-06-15

**Authors:** Wenjie Jiang, Lan Liu, Yueyue Wu, Peng Zhang, Feiyang Li, Juqing Liu, Jianfeng Zhao, Fengwei Huo, Qiang Zhao, Wei Huang

**Affiliations:** Key Laboratory of Flexible Electronics (KLOFE), Institute of Advanced Materials (IAM), Nanjing Tech University (NanjingTech) 30 South Puzhu Road Nanjing 211816 China iamjqliu@njtech.edu.cn iamjfzhao@njtech.edu.cn iamwhuang@njtech.edu.cn; Key Laboratory for Organic Electronics and Information Displays, Institute of Advanced Materials (IAM), SICAM, Nanjing University of Posts & Telecommunications (NUPT) 9 Wenyuan Road Nanjing 210023 China

## Abstract

Room temperature phosphorescent (RTP) materials are rising and gaining considerable attention due to their special photo-capture–release ability. Herein, a kind of environmentally friendly RTP composite was devised by microwaving a mixture of carbon dots, boric acid, and urea, in the presence of covalent bonds and hydrogen bonds between each of the components. The resultant RTP material showed ultra-long phosphorescence lifetime up to 1005.6 ms with an outstanding afterglow as long as 9.0 s. Moreover, this afterglow feature with moisture sensitive behavior was explored to achieve multilevel anti-counterfeiting, with the function of complex decryption of encrypted secret information under multiple stimuli. Our results provide a green strategy for scalable synthesis of carbon-based RTP materials, and extend their application scope to high level information security.

## Abstract 2

A green-synthesized nanocarbon phosphorescent material was applied to multilevel anti-counterfeiting fields, with ultra-long phosphorescence lifetime and humidity sensitivity.

## Graphical abstract

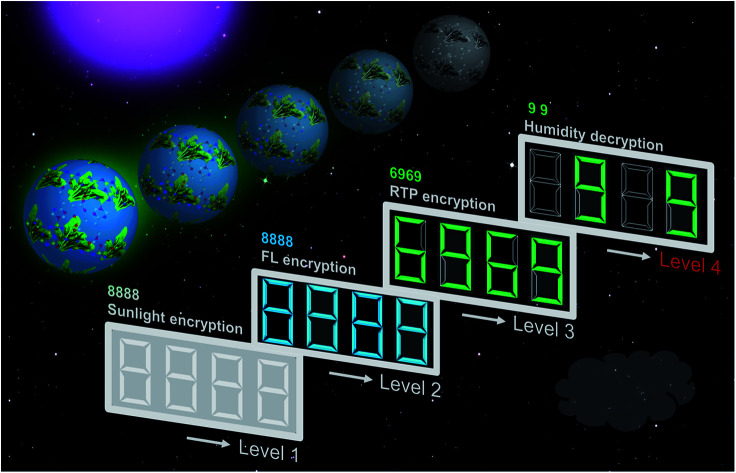

Recently, room temperature phosphorescent (RTP) materials have attracted enormous attention because of their diverse applications in optoelectronics,^1,2^ biochemistry,^3,4^ information encryption/decryption,^5–7^ and anti-counterfeiting.^8^ The development of RTP materials is greatly limited by spin-forbidden transition and non-radiative decay of triplet excitons.^9^ Several kinds of RTP material, mainly including inorganic molecules,^10–12^ organic molecules and polymers,^13–20^ graphene^21,22^ and carbon dots (CD),^23,24^ have been successfully achieved. Among them, the flourishing CD materials exhibited the advantages of unique structure,^25^ simple preparation,^26^ good biocompatibility, tunable electrical and optical properties,^27^ and renewable resources. Since the preparation of the first CD RTP material by Deng's group^28^ by pyrolyzing disodium ethylenediaminetetraacetic acid and polyvinyl alcohol (PVA) in 2013, two main strategies have been explored to construct this kind of material. The first strategy is to add heteroatoms, such as nitrogen,^29–31^ phosphorus,^32^ boron,^33,34^*etc.*, or aromatic carbonyl groups to enhance spin–orbit coupling to boost the efficiency of intersystem crossing (ISC). By utilizing phosphoric acid and ethanolamine as precursors, Jiang^35^*et al.* have prepared carbon-based RTP materials with a visible afterglow to the naked eye. Furthermore, they obtained polymer quantum dot RTP materials from phosphoric acid and ethylenediamine *via* a facile heat treatment.^36^ The second strategy is to stabilize triplet excitons by limiting the molecular vibration and rotation with the help of the matrix or crystals including PVA,^37^ polyurethane,^26^ zeolite,^38,39^ salts,^40,41^*etc.*

Owing to their unique properties, CD RTP materials have demonstrated application potential in several areas.^42–45^ For example, Li^30^*et al.* devised smart anti-counterfeiting inks with nitrogen-doped CD RTP materials which could be painted to encrypt and decrypt complex patterns and information, and the level of information security mostly focuses on single-level anti-counterfeiting. Li^23^*et al.* synthesized CD RTP materials to monitor the concentration of ferric ions *via* a static quenching effect. Liang^3^*et al.* achieved biological imaging successfully owing to the outstanding biocompatibility and low toxicity of a carbon-based RTP composite consisting of silicon dioxide and CD. Although great progress has been made in the research of nanocarbon phosphorescent materials, extending the synthetic methodologies and application ability is highly desirable.

Here, we present a kind of green-synthesized phosphorescent CD composite from spinach-derived CD, boric acid and urea (BNO matrix) using microwave heating. The as-prepared composite (CD/BNO) was characterized by transmission electron microscopy (TEM), X-ray photoelectron spectroscopy (XPS) and Fourier transform infrared spectroscopy (FT-IR). Interestingly, the CD/BNO composite exhibited typical photo-capture-release behavior with phosphorescence (PH) decay under variable light intensity, and the PH phenomenon was systematically investigated by ultraviolet-visible (UV-Vis) and time-resolved fluorescence (FL) spectroscopy. As a proof of concept, the ultralong afterglow was utilized as a security medium to realize high level anti-counterfeiting application.

As illustrated in Fig. 1, the vegetable spinach was transformed into CD by a hydrothermal reaction according to our previous work.^46^ The synthetic CD turned into a CD/BNO composite with the aid of the BNO matrix by microwave treatment. The composite presented as a light yellow solid under daylight at room temperature and emitted blue photoluminescence under a UV lamp of 365 nm. After removal of UV irradiation, a green color afterglow phenomenon was observed. Compared with other previous strategies for the synthesis of afterglow nanocarbon materials, this method provides a simple and green synthetic process.

**Fig. 1:**
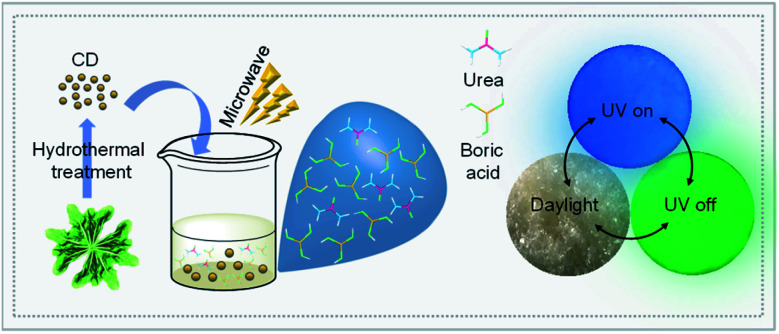
Schematic illustration for the preparation of the CD/BNO composite.

The chemical structure and elemental composition of the CD/BNO composite were investigated. As shown by the TEM image in Fig. 2a, the size of CD with a quasi-spherical shape is 2–5 nm. From measurements of XPS spectra in Fig. 2b, the elemental composition is mainly C (15.04%), N (7.41%), O (45.85%), and B (31.7%), corresponding to the element distribution. The high resolution XPS of B 1s was fitted into four peaks at 191.6 eV, 192.3 eV, 193.0 eV, and 194.2 eV, indicating the presence of B–N, B–C, O–B=O, and B–O within the composite (Fig. 2c). EDS mapping was also performed to prove the existence of elements with different element ratios including C (11.42%), N (12.31%), O (57.64%), and B (18.64%), which were separately decorated on CD/BNO (Fig. 2d).

**Fig. 2:**
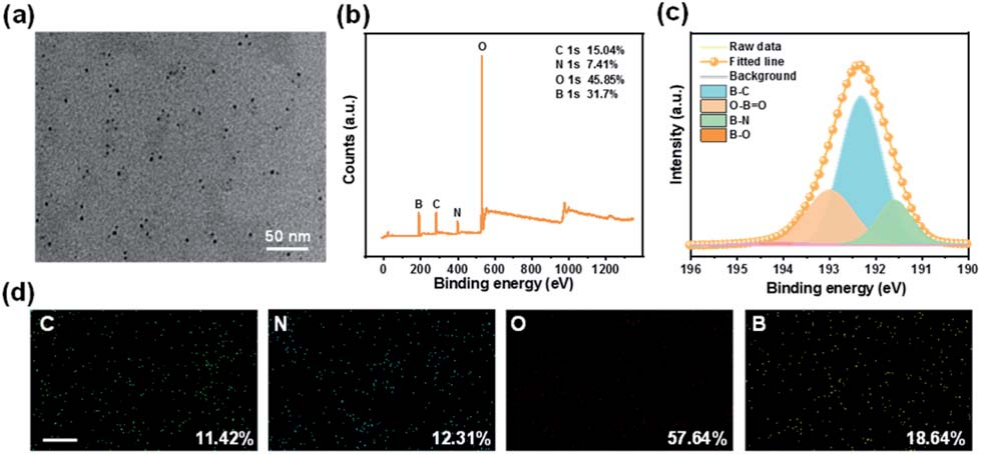
(a) TEM image of CD/BNO. (b) XPS spectra of CD/BNO. (c) XPS high-resolution B 1s spectra of CD/BNO. (d) Energy-dispersive X-ray spectroscopy (EDS) mapping images of CD/BNO, scale bar is 5 μm.

Likewise, the FT-IR displayed three feature peaks of B–N, B–C, and B–O at 780 cm^−1^, 926 cm^−1^, and 1460 cm^−1^, respectively, among which the presence of B–C demonstrated the formation of covalent bonds between CD and BNO. Obviously, the peak of hydrogen bonds in the composite (Fig. 3a, red line) was deeper than that of BNO and CD (blue line and green line) indicating more hydrogen bonds within CD/BNO. By further comparing the XPS high-resolution C 1s spectra of BNO and CD/BNO, it was clearly found that the peak of C=O red-shifted with an increased number of hydrogen bonds in the latter one (Fig. 3b). Fig. 3c showed the schematic illustration for CD encapsulated in the BNO matrix, with the cooperative interaction of supramolecular force and covalent bonds.^21,47^ The right part of Fig. 3c presents the schemed hydrogen bonds (black dotted circles) between different functional groups from the CD and BNO matrix. We assumed that the coexistence of hydrogen bonds and newly formed chemical bonds enhanced their structural rigidity and decreased vibrational dissipation, protecting triplet excitons from being quenched and then bringing about the PH emission.^9,42^

**Fig. 3:**
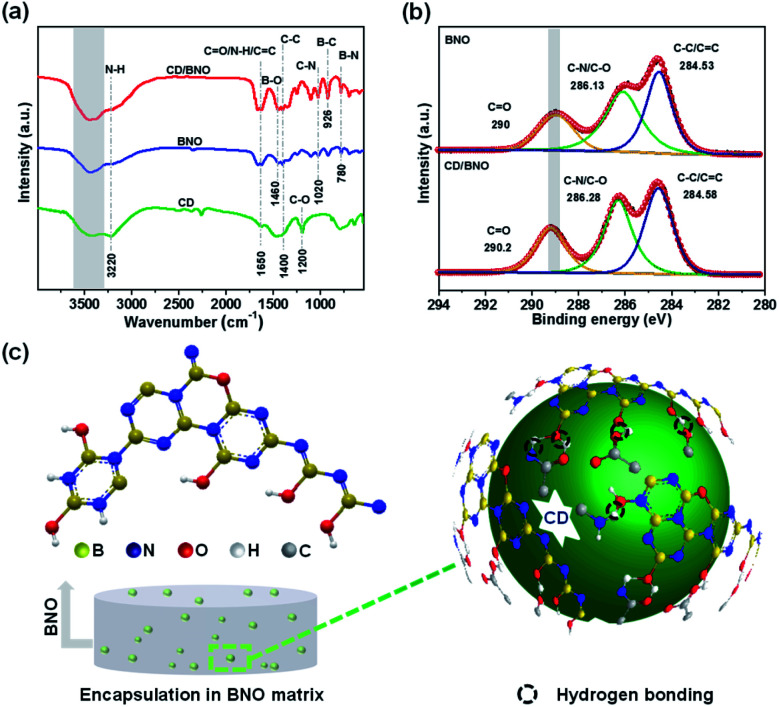
(a) FT-IR spectra of CD/BNO, BNO and CD, respectively. (b) XPS C 1s spectra of CD/BNO and BNO. (c) Possible bonding methods between the BNO matrix and the functional groups on the surface of CD.

The absorption bands of CD mainly located at 270 nm and 320 nm (blue line) owing to the π → π* transition of C=C and n → π* transition of C=O,^48^ respectively (Fig. 4a). The overlap of the PH excitation (PLE) spectrum (red line) and UV-Vis spectrum at 250–350 showed that RTP mainly originates from C=O bonds of CD. The PH emission peaks of CD/BNO and FL emission peaks of the CD and CD/BNO were located at 490 nm, 415 nm, and 425 nm, respectively (Fig. 4b). Generally, the sole CD and nanocarbon composite could emit obvious single FL^49–51^ and RTP, respectively. The strong PH was probably attributed to the newly formed bonds between the CD and BNO matrix. In addition, the PH lifetime of the composite at 475 nm excited by 295 nm is 1005.6 ms (Fig. 4c), which is comparable with that of other carbon- or polymer-based RTP materials.^9,44^

**Fig. 4:**
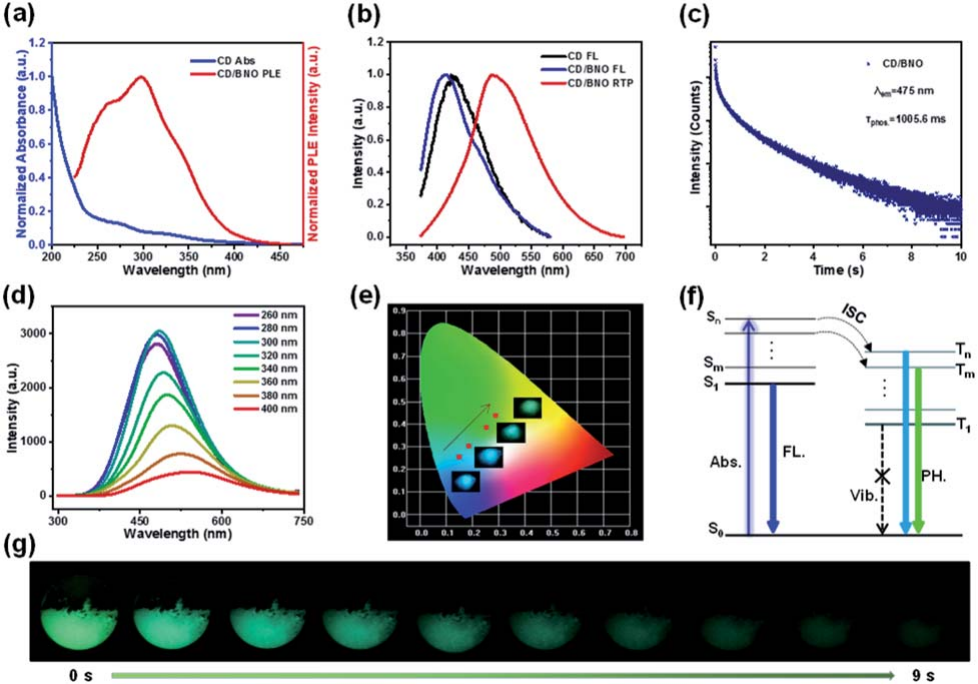
(a) UV-Vis spectrum (blue line) of CD and PLE spectrum of CD/BNO (red line). (b) FL emission spectra of CD and CD/BNO, and PH emission spectrum of CD/BNO, respectively, excited at 300 nm. (c) PH decay curve and lifetimes of CD/BNO monitored at 475 nm with 295 nm excitation. (d) PH spectra of CD/BNO excited at different wavelengths. (e) the changes in color coordinates at four excitation wavelengths were recorded in the Commission International de l'Eclairage (CIE) coordinate chart. The inset shows the ultra-long luminescence photos of the powder taken after excitation at 280, 300, 350, and 400 nm, respectively. (f) Energy level diagram of the nanocarbon composite (Abs.: absorption; Vib.: vibration). (g) PH emission images after ceasing the UV lamp (365 nm) irradiation for 0 s to 9 s under ambient conditions.

Fig. 4d shows that the composite emits variable fluorescence from blue to green depending on the excitation wavelength.^52^ This trend was further confirmed by the color coordinates with inset optical images under different excitation wavelengths (Fig. 4e). The proposed mechanism of the excitation dependence has three processes (Fig. 4f). Firstly, the excitons transition from the ground state (S_0_) to different singlet excited states. Then, the excitons approach different triplet excited states through ISC. Finally, they return to S_0_ with variable PH colors induced by the aforementioned photophysical process. Impressively, the duration time of the visible afterglow could reach as long as 9 s (*λ*_ex_ = 365 nm) measured by a digital camera (Fig. 4g).

To assess the influence of external environment on PH properties, the time and intensity of the afterglow were tested under different external stimuli, such as humidity, irradiation time, and temperature. By placing power under variable humidity conditions for one hour, the time of the afterglow gradually decreased with the increase of humidity (Fig. 5a). The composite absorbs water molecules under moisture due to the hydrophilicity of BNO, and the oxygen in water leads to the shortening of PH time. As shown in the PH part of the Jablonski diagram (Fig. 5b),^53^ paramagnetic oxygen makes the luminophore approach the triplet excited state through the ISC process. Meanwhile, molecular oxygen rises to different excited states and changes back to ^3^∑^−^_g_. The singlet oxygen is generated in the process, and the quantum yield approaches unity.^53^ Therefore, triplet excitons are easily quenched by oxygen, which leads to less triplet excitons returning to S_0_ with weak and short time PH. This oxygen-induced quenching feature provides a useful tool to construct multiple information encryption and anti-counterfeiting. Furthermore, irradiation time and external temperature also influence PH intensity. With the increase of irradiation time, the intensity and time of the afterglow enhance until saturation (Fig. 5c). The inner small image is a magnification of the blue dashed box, which can be more clearly seen. Fig. 5d shows the decay curves under distinct temperature factors with the same irradiation time of 4 s. Following the increased temperature, the intensity gradually evolved with decremental trends, which was ascribed to the mechanism that excitons move to a higher nonradiative vibrational energy level till point *E* (spillover point) at higher temperature, thus leading to less triplet excitons with weaker PH.

**Fig. 5:**
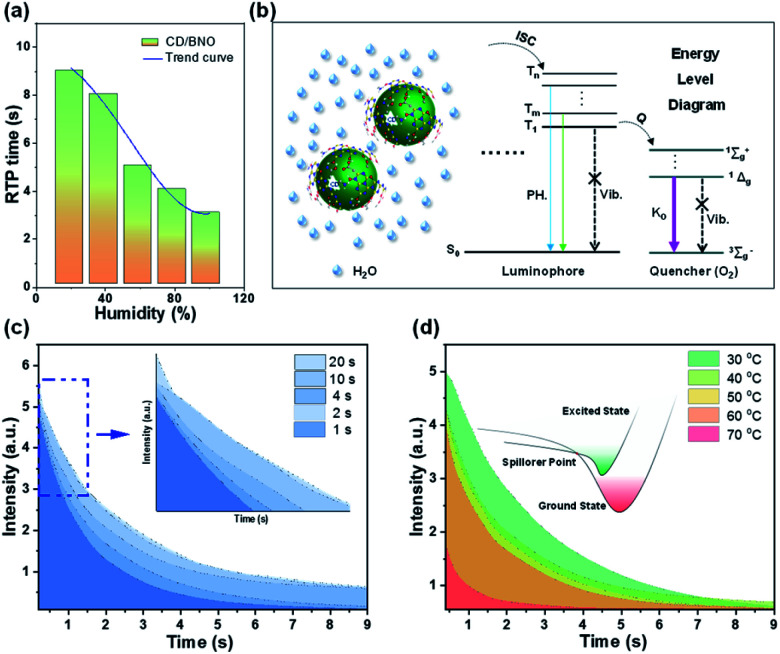
(a) PH time in response to humidity. (b) The influence mechanism of humidity on PH time (Q: quenching; ^1^Δ_g_: the first excited state; ^1^∑^+^_g_: the second excited state; ^3^∑^−^_g_: triplet ground state; K_O_: singlet oxygen). (c) The response of PH time to the duration of excitation with the light source. (d) The response of PH time to external temperature.

Since the CD/BNO composite exhibits unique afterglow properties with moisture sensitivity, the material is a promising information carrier for advanced anti-counterfeiting technology. As a proof-of-concept application, the complex encryption/decryption of CD/BNO was studied. By rationally designing a multilevel encryption protocol with a 4 × 7 bar-shaped pixel matrix fulfilled by CD/BNO, CD phosphor and NOA63 (Fig. 6a), the synthetic CD/BNO and CD blue phosphor^50^ were placed in the position of numbers “6” and “9”, respectively. Meanwhile, the two number “9” were covered with transparent commercial NOA63 film, and the two number “6” were exposed to air. Like this, a piece of number information “99” (Fig. 6b) was encrypted into the pixel matrix as four white “8888” without other observable number information. Then several different stimuli including sunlight, UV on, UV off, and humidity would be exerted in sequence for the decryption of target “99”. Under sunlight, wrong information of “8888” was observed, thus the first level decryption failed (Fig. 6c). When UV light was turned on, blue “8888” was still there as the error message (Fig. 6d) owing to the same fluorescence of CD/BNO and blue phosphor. After turning off the UV light, fake information “6969” was observed on the pixel matrix due to the ultralong afterglow of the CD/BNO composite. Finally, when the pixel matrix was placed in a wet environment for four hours and removed from the UV excitation, the true information “99” was decrypted from “8888”, because the unencapsulated CD/BNO parts can quickly absorb moisture and the absorbed oxygen quenches the phosphorescent triplet excitons.^53^ The two encapsulated “9” were isolated from moisture, so PH emission can still be maintained. It is concluded that UV light of 365 nm and humidity are the two key factors for the decryption of “99” from the “8888”.

**Fig. 6:**
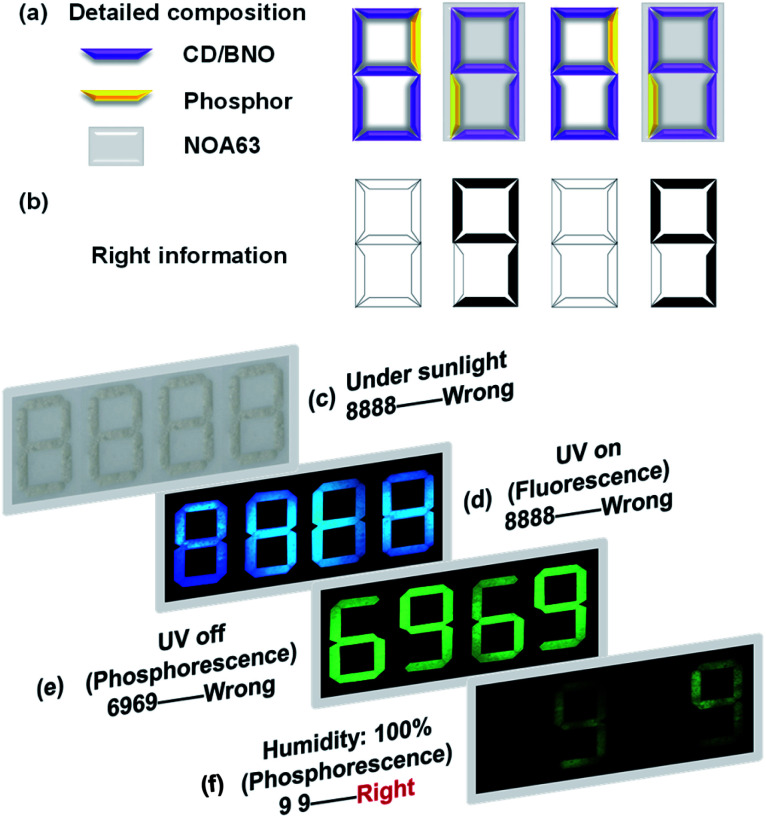
(a) Detailed composition of the patterned template. (b) Hidden right information. (c) Digital photos of a 4 × 7 bar-shaped pixel matrix fulfilled by CD/BNO, CD phosphor and NOA63 (Norland Optical Adhesive 63) under sunlight. (d) Digital photos with UV on. (e) Corresponding images with UV off in different environments. (f) Demonstration of the anti-counterfeiting.

## Conclusions

In summary, we have demonstrated a green-synthesis of an RTP carbon dot composite from spinach-derived CD, boric acid and urea. The existence of hydrogen bonds and newly formed covalent bonds among each of the components increased the rigidity of the structure while reducing the vibration and rotation of the molecules, resulting in PH emission. The obtained composite showed an ultra-long PH lifetime of 1005.6 ms and an obvious afterglow emission of 9.0 s. Moreover, the time and intensity of the afterglow phenomenon are strongly affected by external stimuli, including moisture, irradiation time, and temperature; thus the composite with such advances was innovatively applied to multilevel anti-counterfeiting with complex information encryption and decryption. Our work provides a new pathway for sustainable production of nanocarbon phosphorescent materials and application insight into multilevel anti-counterfeiting fields.

## Conflicts of interest

There are no conflicts to declare.

## Supplementary Material

NA-003-D1NA00252J-s001
