# Supplementary material for: A green-synthesized phosphorescent carbon dot composite for multilevel anti-counterfeiting
Source: Nanoscale Adv. 2021 Jun 15;3(15):4536–40. doi: 10.1039/d1na00252j (PMC9419446; doi:10.1039/d1na00252j)
Supplement: NA-003-D1NA00252J-s001 [file NA-003-D1NA00252J-s001.pdf]

Electronic Supplementary Material (ESI) for Nanoscale Advances.
This journal is © The Royal Society of Chemistry 2021

Supporting information

Green-Synthesized Phosphorescent Carbon Dot Composite for Multilevel Anti-Counterfeiting

Wenjie Jiang,[a] Lan Liu,[a] Yueyue Wu,[a] Peng Zhang,[a] Feiyang Li,[b] Juqing Liu,*[a] Jianfeng Zhao,*[a] Fengwei Huo,[a] Qiang Zhao,[b] & Wei Huang*[ab]

[a]Key Laboratory of Flexible Electronics (KLOFE) & Institute of Advanced Materials (IAM), Nanjing Tech University (NanjingTech), 30 South Puzhu Road, Nanjing 211816, China.
E-mail: iamjqliu@njtech.edu.cn; iamjfzhao@njtech.edu.cn; wei-huang@njtech.edu.cn

[b]Key Laboratory for Organic Electronics and Information Displays & Institute of Advanced Materials (IAM), SICAM, Nanjing University of Posts & Telecommunications, 9 Wenyuan Road, Nanjing 210023, China.

Experimental Section

Materials

Boric acid (BA, ≥99.5%) and urea (ODE, ≥99.0%) are purchased from Sinopharm Chemical Reagent Co., Ltd. Dialysis bag (1k Da) is purchased from MYM Biological Technology Company. Anhydrous oxalic acid (CA, ≥97%) and octadecene (ODE, ≥90%) are purchased from Tiexi (Shanghai) chemical industry development. Ethanol (≥99.7%) is purchased from Wuxi City Yasheng Chemical Corporation. UV LED chip is purchased from Guangsheng Semiconductor Company.

Preparation of spinach-derived carbon dots

According to our group's previous work, we used spinach as self-doped carbon sources purchased from local markets. After putting 100 g precursor and 20 mL deionized water in an autoclave at 180 ^{o}C for 12 hours, the crude product was obtained. The reaction solution was roughly filtered with a 0.22 μm filter membrane, and then transferred to a 1kDa dialysis bag for dialysis. The spinach-derived carbon dots (CD) were purified through dialysis membrane (Da = 1000) against deionized water for 7 days. The solvent was changed every 12 hours in the first three days and every 24 hours in the next four days.

Preparation of phosphor

After the 15 mL octadecene and 1.5 g hexadecylamine were reacted in a nitrogen atmosphere for 1.5 hours, 1.8 g oxalic acid was added to the three-necked flask to continue the reaction for 3 hours. Finally, the product was washed seven times to obtain blue phosphor.

Preparation of CD/BNO composite

The target product was obtained by one-step microwave reaction. Typically, the mixture of 0.6 g boric acid, 0.3 g urea, and 150 μL spinach-derived CD was dissolved in 10 mL deionized water, respectively. Then, the reactants were placed in a microwave oven for 10 minutes with the power of 700 W. Finally, the powder which attached to the wall of the beaker was the target product.

Preparation of anti-counterfeiting application

Firstly, the CD/BNO (encryption material) was placed in the "6" and "9" in the patterned polytetrafluoroethylene pixel matrix (background material) "8" and blue phosphor (interfering material) in the small "1". Secondly, the second and fourth numbers was covered tightly with a transparent film (NOA63). Thirdly, the moisture absorption treatment for the pattern was carried out by placing it in an environment of 100% humidity for four hours. The procedures above were the preparation method of multilevel encryption and decryption.

Characterization

Transmission electron microscopy (TEM) images were taken on a HITACHI 7605 microscope. The content of elements was measured by scanning electron microscope (SEM, JSM 7800F). The photoluminescence spectra were recorded by a Fluorescence Spectrometer (F-4600) and UV-Vis absorption spectra were measured with a Shimadzu UV-1750 Spectrophotometer. X-ray photoelectron spectroscopy (XPS, Thermo Escalab 250Xi) was used to confirm elements and chemical composition of material. Fourier Transform Infrared Spectroscopy (FTIR, DT-40) spectra were measured by neat on a KBr plate. The lifetime was taken on an Edinburgh FLSP920 fluorescence spectrophotometer equipped with a xenon arc lamp (Xe900) and a microsecond flash-lamp (μF900). Luminescent photos were taken by VARIO-SUMMILUX-H 1:1.8-3.4/18-125 ASPH.
